# Fetal calf serum and retinoic acid affect proliferation and terminal differentiation of a rat rhabdomyosarcoma cell line (BA-HAN-1C).

**DOI:** 10.1038/bjc.1989.12

**Published:** 1989-01

**Authors:** C. D. Gerharz, H. E. Gabbert, H. K. Biesalski, R. Engers, C. Luley

**Affiliations:** Department of Pathology, Johannes Gutenberg-University of Mainz, Federal Republic of Germany.

## Abstract

**Images:**


Br. J. Cancer (1989), 59, 61–67 © The Macmillan Press Ltd., 1989

### Fetal calf serum and retinoic acid affect proliferation and terminal differentiation of a rat rhabdomyosarcoma cell line (BA-HAN-1C)

C.D. Gerharz[1], H.E. Gabbert[1], H.K. Biesalski[2], R. Engers[1] & C. Luley[3]

*Departments of [1]Pathology, [2]Biochemistry and [3]Internal Medicine, Johannes Gutenberg-University of Mainz, D-6500 Mainz, Federal Republic of Germany.*

**Summary** We report on the establishment of a model for differentiation induction in sarcomas, using the clonal rhabdomyosarcoma cell line BA-HAN-1C. This rhabdomyosarcoma cell line is composed of morphologically undifferentiated mononuclear stem cells, some of which spontaneously fuse to form terminally differentiated multinuclear myotube-like giant cells. The deprivation of fetal calf serum (FCS) or the exposure to retinoic acid, respectively, resulted in a significant inhibition of proliferation ($P<0.001$) and a marked increase in cellular differentiation as shown by a significant increase in the number of myotube-like giant cells ($P<0.001$) and in the creatine kinase activity ($P<0.05$) used as a biochemical marker of differentiation. Furthermore, after exposure to retinoic acid about 30% of the mononuclear tumour cells exhibited morphological features of rhabdomyogenic differentiation, such as bundles of thick and thin myofilaments, which had never been observed in the mononuclear cells of untreated cultures. These results confirm that the inverse linkage between proliferation and differentiation known from embryonic myogenesis is preserved in our rhabdomyosarcoma cell line. The failure to induce terminal differentiation by exposure to retinoic acid in all the cells of our clonal cell line indicates that some tumour cells might epigenetically be blocked from responding to retinoic acid. The temporary growth retardation observed after FCS-deprivation suggests that autocrine stimulation of proliferation may be operating in our cell line, too.

Cancer cells have not necessarily lost all the genes that control proliferation and differentiation. Thus, in many cancers at least some of the tumour cells exhibit abortive attempts at normal differentiation, sometimes proceeding to terminally differentiated post-mitotic cells, as was shown for myeloid leukaemias, squamous cell carcinomas, teratocarcinomas, neuroblastomas and rhabdomyosarcomas (Pierce, 1974*a*,*b*; Dexter *et al*., 1977; Strickland & Mahdavi, 1978; Linder *et al*., 1981; Moore *et al*., 1986; Paukovits *et al*., 1986; Sidell *et al*., 1986; Sachs, 1987; Gabbert *et al*., 1988). These observations stimulated attempts to induce differentiation as an alternative to standard cytotoxic chemotherapy (for review see Freshney, 1985).

Sarcomas have received less attention, partly because cell type specific markers of differentiation are not easily available in many established sarcoma cell lines. We now report the establishment of a new model for differentiation induction in sarcomas, using the clonal rhabdomyosarcoma cell line BA-HAN-1C, which was derived from a dimethylbenzanthracene-induced rhabdomyosarcoma in rat (Gerharz *et al*., 1988). This clonal rhabdomyosarcoma cell line BA-HAN-1C closely imitates embryonic rhabdomyogenesis and is composed of myogenically committed but morphologically undifferentiated mononuclear stem cells, some of which fuse to form multinuclear myotube-like giant cells with morphological features of rhabdomyogenic differentiation, such as bundles of thick and thin myofilaments. Furthermore, the mitotic activity in our rhabdomyosarcoma cell line is entirely confined to the mononuclear cell compartment, whereas the myotube-like giant cells have irreversibly withdrawn from the mitotic cycle and represent terminally differentiated post-mitotic cells (Gabbert *et al*., 1988). In this report, we show that fetal calf serum and retinoic acid are able to affect the proliferation and differentiation of our clonal rhabdomyosarcoma cell line BA-HAN-1C, and that the control mechanisms of proliferation and differentiation known from embryonic myogenesis are not completely lost in this cell line.

### Material and methods

### *Cells and culture*

The clonal cell line BA-HAN-C1 was derived from a dimethylbenzanthracene-induced rhabdomyosarcoma in rat (Gerharz *et al*., 1988). The clonal origin of this cell line had been confirmed by repeated cloning procedures and investigations were performed with cultures between passage numbers 10 and 30. The standard growth medium was Dulbeccos's modified Eagle medium (DMEM, Gibco Europe, FRG), supplemented with 10% heat-inactivated fetal calf serum, penicillin and streptomycin. The same batch of FCS was used for all experiments. Unless otherwise noted, cultures were refed after four days. The tumour cells were cultured in Nunclon®-flasks (Gibco Europe, FRG) and incubated in an atmosphere with 5% $CO_2$ at 37°C.

Received 3 June 1988; and in revised form, 22 September 1988.

### *Induction of differentiation*

*FCS depletion* Twenty-four hours after plating of BA-HAN-1C tumour cells, the standard growth medium with 10% FCS was substituted by growth medium with a reduced FCS-concentration (1% and 5%).

*Retinoic acid treatment* A stock solution of 5 mM retinoic acid (Serva, FRG) was prepared in 95% ethanol. Preliminary experiments showed that concentrations of retinoic acid exceeding 5 μM heavily depressed tumour cell proliferation and resulted in a detachment of the adherent tumour cells as well as a reduced viability, evidenced by the trypan blue exclusion test. For differentiation induction, the stock solution was diluted in standard growth medium to a concentration of 0.1 μM and 1 μM. Prior to the experiments, the purity of the commercial standard and the calculated concentrations of retinoic acid were checked using HPLC (Annesley *et al*., 1984; Biesalski & Hafner, 1988). The HPLC-analysis demonstrated a ratio 13-*cis*:all-*trans* retinoic acid of 1:3. Further derivates were not detected. The total vitamin A concentration in the normal standard medium supplemented with 10% FCS was 0.3 nM. Twenty-four hours after plating of BA-HAN-1C cells, the standard growth medium was substituted by growth medium supplemented with retinoic acid. For light protection, the culture flasks were wrapped in aluminium foil.

Solvent controls (0.02% ethanol) produced no effects on BA-HAN-1C tumour cell growth or differentiation.

### *Assessment of differentiation* in vitro

In vitro *morphology* For scanning electron microscopy and transmission electron microscopy the tumour cells were seeded on glass coverslips. After incubation for one week, the tumour cells were fixed *in situ* and further handled as described previously (Gerharz *et al.*, 1988).

*Fusion assay* Some $3 \times 10^5$ BA-HAN-1C tumour cells each were seeded into $25 cm^2$ culture flasks. On the bottom of these culture flasks four arbitrarily located fields had been marked. The area marked out by these four fields was 1/32 the growth area of the culture flask. After 24 hours the standard growth medium was completely substituted by the differentiation-inducing media. The number of myotube-like giant cells in the marked fields was counted by phase contrast microscopy after 24 and 96 hours. Cells that contained three or more nuclei were classified as myotubes. The effects of FCS-depletion (1% and 5%) or retinoic acid (0.1 $\mu$M and 1 $\mu$M) were evaluated in five replicate culture flasks per concentration. As a control, the frequency of myotube-like giant cells was determined in five culture flasks each with standard growth medium or standard growth medium with 0.02% ethanol, respectively. At the end of the observation period of 96 hours, the total number of tumour cells was determined in each culture flask. Because the formation of myotube-like giant cells by fusion is affected by cell density, the effects of different culture conditions on cell proliferation and cell density had to be compensated. To this end, the relative frequency of myotube-like giant cells, i.e. the ratio between the number of myotube-like giant cells and the total number of cells per culture flask, was calculated.

*Creatine kinase activity* Triplicate samples of $5 \times 10^6$ cells grown for ten days in growth medium with 1% FCS or growth medium with 1 $\mu$M retinoic acid, respectively, were disrupted by sonication. After centrifugation, the total creatine kinase activity, which was used as a biochemical differentiation marker (Delaporte *et al.*, 1986; Garvin *et al.*, 1986), was determined at 37°C on an Olympus AU 5031 analyser using the CK-test (NAC-activated) from Merck (Darmstadt, FRG).

### *Assessment of growth properties* in vitro

*Growth rate* Fifteen replicate $25 cm^2$ culture flasks were each exposed to differentiation-inducing medium. As a control, 15 replicate $25 cm^2$ flasks were each exposed to standard growth medium and standard growth medium supplemented with 0.02% ethanol, respectively. Each culture flask was seeded with $5 \times 10^4$ cells. In each experiment, cells from three culture flasks were harvested separately each day for five days and haemocytometer cell counts with the Neubauer chamber were performed. Cells were not refed during this period.

*Plating efficiency* Tumour cells were seeded onto triplicate 96-microwell plates (Gibco Europe, FRG) at definite concentrations in differentiation-inducing media and incubated for 2 weeks without refeeding. The cells had been pretreated for one week by exposure to either FCS-depleted growth medium (1% FCS) or growth medium supplemented with 1 $\mu$M retinoic acid. After 14 days, the plating efficiency was determined by counting the total number of colonies and relating them to the control.

### Results

### *Effects of FCS on cellular differentiation*

In vitro *morphology* No phenotypic difference was evident in FCS-depleted cultures by phase contrast microscopy and scanning electron microscopy (Figure 1c and d) when compared to controls (Figure 1a and b) in standard growth medium. The ultrastructural characteristics of both the mononuclear cells and the myotube-like cells closely corresponded to their counterparts in standard growth medium.

*Fusion assay* FCS-deprivation resulted in a marked dose-dependent increase in the absolute number of myotube-like giant cells after 96 hours in culture. The relative frequency of myotube-like giant cells, i.e. the ratio between the number of myotube-like giant cells and the total number of cells per culture flask, exhibited a statistically significant ($P < 0.001$) dose-dependent increase. (See Table I.)

*Total creatine kinase activity* Exposure of BA-HAN-1C tumour cells to growth medium supplemented with 1% FCS for 10 days resulted in a statistically significant ($P < 0.05$) increase in the total creatine kinase activity, which was used as a biochemical marker of differentiation. (See Table II.)

### *Effects of FCS on proliferation* in vitro

*Growth rate* Under the conditions of our experiments, FCS deprivation resulted in a statistically significant ($P < 0.001$) temporary retardation of growth. (See Figure 2.) After three days in culture, the cell density of FCS-depleted culture flasks ($7.9 \times 10^5 \pm 5 \times 10^4$ cells per culture flask) remained below the cell density of the control ($1.7 \times 10^6 \pm 2.4 \times 10^5$ cells per culture flask). Detrimental effects of FCS-depletion on cell viability could be excluded by the trypan blue exclusion test. After three days in culture, BA-HAN-1C cells exhibited a mean doubling time of 16 hours that did not differ between FCS-depleted growth medium and standard growth medium. The initial growth retardation by FCS-deprivation was compensated for during the next days in culture by a delayed plateau phase of growth when compared to the control. Thus, after 7 days in culture the cell density per culture flask did not significantly differ between FCS-depleted culture flasks ($3.9 \times 10^6 \pm 2.5 \times 10^5$ cells per culture flask) and control flasks ($4.7 \times 10^6 \pm 7.2 \times 10^5$ cells per culture flask).

*Plating efficiency* Pretreatment with FCS-depleted growth medium for 1 week and plating in FCS-depleted growth medium markedly affected the plating efficiency, prohibiting any colony formation at cell concentrations of up to 10 cells per microwell. (See Table III.)

### *Effects of retinoic acid on cellular differentiation*

In vitro *morphology* Seventy-two hours after exposure to medium supplemented with 1 $\mu$M retinoic acid, the mononuclear cells of BA-HAN-1C appeared to be more elongated and spindle-shaped by phase contrast microscopy and scanning electron microscopy (Figure 1e and f). In confluent cultures the cells were closely aligned side-by-side in a more orderly arrangement and piling up was significantly less evident when compared to the criss-crossed growth pattern under standard growth conditions (Figure 1a and b). Transmission electron microscopy showed that about 30% of the mononuclear tumour cells (Figure 3b, c and d) exhibited irregular bundles of thick myofilaments (12–15 nm in diameter) and thin myofilaments (6–8 nm in diameter), i.e. features of rhabdomyogenic differentiation that had never been observed in their mononuclear counterparts (Figure 3a) under standard growth conditions. Substantial amounts of monoparticulate glycogen deposits (Figure 3e) were found in these mononuclear cells, too, and some mononuclear cells contained networks of T-system-like tubules (Figure 3f). The ultrastructural characteristics of myotube-like giant cells did not differ from those of their multinuclear counterparts under standard conditions.

*Fusion assay* Exposure to retinoic acid resulted in a marked dose-dependent increase in the absolute number of myotube-like giant cells after 96 hours in culture. The relative frequency of myotube-like giant cells, i.e. the ratio between the number of myotube-like giant cells and the total number of cells per culture flask, exhibited a statistically significant ($P < 0.001$) dose-dependent increase. (See Table I.)

**Figure 1** Scanning electron microscopy and phase contrast microscopy of BA-HAN-1C cells grown for one week in standard growth medium (**a**, **b**), in medium supplemented with 1% FCS (**c**, **d**), or with 1 μM retinoic acid (**e**, **f**): small mononuclear cells exhibiting a criss-crossed growth pattern in standard growth medium (**a**) and FCS-depleted medium (**c**) as opposed to the more regular arrangement in medium supplemented with retinoic acid (**e**). Abundant myotube-like giant cells (arrows) in FCS-depleted medium (**d**) and in medium supplemented with retinoic acid (**f**) as opposed to sparsely distributed myotube-like giant cells (arrow) in standard growth medium (**b**). **a**–**f**: bar = 20 μm.

**Table I** Fusion assay of BA-HAN-1C tumour cells in FCS-depleted medium and in medium supplemented with retinoic acid

| | *Number of myotube-like giant cells*[a] | | *Ratio* $\frac{\text{number of myotube-like giant cells}^{a} \times 10^4}{\text{total number of cells}}$ | |
|---|---|---|---|---|
| | *Initially* | *After 96 hours* | *Initially* | *After 96 hours* |
| Control | 14.7±1.2 | 75±7.2 | 0.49±0.04 | 0.10±0.01 |
| 5% FCS | 9.3±2.1 | 365±216 | 0.31±0.07 | 0.51±0.31 |
| 1% FCS | 11.0±5.6 | 1741±543 | 0.36±0.19 | 3.57±0.95 |
| Control | 4.8±2.2 | 32.8±2.2 | 0.16±0.07 | 0.04±0.00 |
| 0.1 μM retinoic acid | 5.8±2.5 | 234±80 | 0.19±0.08 | 0.30±0.13 |
| 1 μM retinoic acid | 5.2±1.8 | 2301±297 | 0.17±0.06 | 3.09±0.33 |

[a]In 1/32 the growth area of a culture flask. Each value represents the mean ± standard deviation of five replicate experiments. The dose-dependent increase of the ratio between the number of myotube-like giant cells and the total number of cells is highly significant ($P<0.001$; analysis of variance with two independent factors, repeated measurements in one factor, i.e. time).

*Total creatine kinase activity* Exposure of BA-HAN-1C tumour cells to growth medium supplemented with 1 μM retinoic acid for 10 days resulted in a statistically significant ($P<0.05$) increase in the total creatine kinase activity used as a biochemical marker of differentiation.

*Effects of retinoic acid on proliferation* in vitro

*Growth rate* Under the conditions of our experiment, exposure to retinoic acid resulted in a statistically significant ($P<0.001$) inhibition of proliferation. (See Figure 4.) Detri-

**Table II** Creatine kinase activity of BA-HAN-1C tumour cells after 10 days in standard growth medium (control), FCS-depleted medium and in medium supplemented with retinoic acid

| | *Creatine kinase activity (mU/5 x 10⁶ cells)* |
|---|---|
| Control | $120 \pm 15$ |
| Medium supplemented with 1% FCS | $1290 \pm 50$ |
| Medium supplemented with 1 μM retinoic acid | $7090 \pm 1060$ |

Each value represents the mean ± standard deviation of three replicate samples. The difference was statistically significant ($P < 0.05$; Wilcoxon test for unpaired samples).

**Figure 2** Growth curves of BA-HAN-1C in standard growth medium (control) and in medium supplemented with 1% FCS. Each value represents the mean of three replicate samples ± standard deviation. The difference between the growth curves is statistically significant ($P < 0.001$; analysis of variance with two independent factors). **a–b**: mean doubling time of 16 hours.

**Table III** Plating efficiency of BA-HAN-1C tumour cells in per cent of the control after exposure to FCS-depleted medium and medium supplemented with retinoic acid

| | *Plating efficiency* | |
|---|---|---|
| | *Growth medium supplemented with* | |
| *Number of cells seeded per microwell* | *1% FCS* | *1 μM retinoic acid* |
| 10 | 0% | 54% |
| 1 | 0% | 3% |
| 0.3 | 0% | 4% |

mental effects of retinoic acid on cell viability could be excluded by the trypan blue exclusion test. After 3 days, BA-HAN-1C cells exposed to retinoic acid exhibited a mean doubling time of 17 hours as compared to a mean doubling time of about 16 hours under standard growth conditions. The prolongation of the mean doubling time by exposure to retinoic acid became increasingly evident after the third day in culture. As a result, the cell density per culture flask during the plateau phase of growth after 7 days differed markedly between the control ($6.4 \times 10^6 \pm 2.8 \times 10^5$ cells per culture flask) and cultures exposed to retinoic acid ($1.1 \times 10^6 \pm 1.9 \times 10^5$ cells per culture flask).

*Plating efficiency* Pretreatment with retinoic acid for one week and plating in medium supplemented with retinoic acid markedly decreased the plating efficiency when compared to the control. (See Table III.)

### Discussion

FCS is known to affect growth and cellular differentiation of non-neoplastic myoblast cell lines derived from embryonic skeletal muscle cells (Scarpa *et al.*, 1975; Königsberg, 1977; Yaffe & Saxel, 1977; Dollenmeier & Eppenberger, 1983; Pinset & Whalen, 1983). Therefore, we investigated the effects of FCS on a rhabdomyosarcoma cell line. Our results show that FCS-deprivation results in a marked initial retardation of proliferation (Figure 2) and in a simultaneous induction of differentiation as indicated by the increase in the number of myotube-like giant cells (Table I) and in the creatine kinase activity (Table II). These effects may be explained by the concept of an autocrine stimulation of proliferation, proposed by Sporn and Todaro (1980). According to this concept, cancer cells do not proliferate absolutely autonomously but in response to polypeptide growth factors, which are constitutively synthesised by the tumour cells (De Larco & Todaro, 1978; Todaro *et al.*, 1980; Marquardt *et al.*, 1983). Autocrine stimulation of tumour cell proliferation, however, can be bypassed with exogeneous growth factors supplemented with calf serum. Thus, fetal calf serum has been shown to contain potent polypeptide growth factors such as PDGF, EGF and TGF-$\beta$ (Alexander, 1985; Heldin *et al.*, 1985, 1986; Florini *et al.*, 1986; Harris *et al.*, 1986; Sporn *et al.*, 1987). Consequently, FCS-deprivation in tissue culture media results in growth retardation, as could also be seen in our rhabdomyosarcoma cell line. However, the proliferation of our rhabdomyosarcoma cell line in FCS-depleted medium (Figure 2) markedly accelerated after 3 days in culture, when a minimum cell density had been achieved, which possibly provided a sufficient concentration of autocrine growth factors. The temporary retardation of growth observed after FCS-deprivation was accompanied by an increase in the proportion of terminally differentiated myotube-like giant cells. This observation suggests that the inverse linkage between proliferation and differentiation, which is known from normal embryonic myogenesis (Nadal-Ginard, 1978), has been preserved in our rhabdomyosarcoma cell line.

Retinoic acid is known to affect the proliferation and differentiation of both non-neoplastic and neoplastic cells (Lotan, 1979; Sporn & Roberts, 1983; Chytil, 1986; Lippmann *et al.*, 1987*a*,*b*), sometimes even converting tumour cells to terminally differentiated postmitotic cells (Strickland & Mahdavi, 1978; Linder *et al.*, 1981; Sherman *et al.*, 1985; Garvin *et al.*, 1986; Paukovits *et al.*, 1986; Sidell *et al.*, 1986). Our results show that retinoic acid inhibits proliferation and simultaneously induces terminal differentiation in our rhabdomyosarcoma cell line. After exposure to retinoic acid, the proliferation of BA-HAN-1C cells was significantly inhibited, and the tumour cells exhibited a more orderly arrangement with less piling up in confluent cultures

**Figure 3** Transmission electron microscopy of BA-HAN-1C cells: mononuclear tumour cells in standard growth medium (**a**) lacking rhabdomyogenic features of differentiation. Mononuclear cell after exposure to 1 $\mu$M retinoic acid for one week (**b**) exhibiting an extensive cytoplasmatic area (star) with irregular bundles of thick and thin myofilaments shown in more detail in **c** and **d**. Monoparticulate glycogen deposits (**e**) and T-system-like tubules (**f**) in a mononuclear cell after exposure to retinoic acid. **a**, **b**, bar = 2 $\mu$m; **c**–**f**: bar = 0.4 $\mu$m.

(Figure 1e), suggesting a partial restoration of the contact inhibition of proliferation after exposure to retinoic acid (Lotan, 1980). The effects of retinoic acid on proliferation were paralleled by a statistically significant increase in the frequency of terminally differentiated post-mitotic myotube-like giant cells (Table I) and an increase in the creatine kinase activity used as a biochemical marker of differentiation (Table II). Furthermore, about 30% of the mononuclear tumour cells exhibited morphological features of rhabdomyogenic differentiation, which had never been observed in the mononuclear tumour cells of untreated cultures (Figure 3). We did not succeed, however, in converting all the cells of BA-HAN-1C into terminally differentiated post-mitotic myotube-like giant cells after exposure to retinoic acid. In our clonal cell line BA-HAN-1C, the coexistence of diverse subpopulations is not very likely to account for this partial refractoriness to retinoic acid as was suggested for other tumour models (Sherman *et al.*, 1986; Zile *et al.*, 1987). Therefore, BA-HAN-1C tumour cells might in some way be epigenetically blocked from responding to retinoic acid, as was discussed for an embryonal carcinoma cell line by Sherman *et al.* (1986).

All things considered, there is an overlap in the effects of FCS-deprivation and exposure to retinoic acid. The effects of FCS-deprivation on the proliferation and differentiation of our rhabdomyosarcoma cell line BA-HAN-1C suggested that polypeptide growth factors not yet chemically defined might be active in our cell line, too, stimulating proliferation in an autocrine manner. On the other hand, retinoic acid was recently shown to modulate the effects of polypeptide growth factors and the actions of oncogenes or the proteins encoded by these oncogenes (Jetten, 1980; Craig *et al.*, 1984; Amatruda *et al.*, 1985; Thiele *et al.*, 1985; Bentley & Groudine, 1986). Therefore, our clonal cell line should provide a useful system for further investigations concerning the interrelations between polypeptide growth factors and retinoic acid, both of which affect the proliferation and differentiation of tumour cells.

We would like to express our appreciation to Mrs A. Niederauer, Mrs K. Molter, Mrs Dellee-Krippes, Mrs C. Bürkner, Mrs B. Häusermann as well as to Mr K. Weber and Mr W. Meyer for their excellent technical assistance. We are grateful to Dr K.H. Schicketanz for his statistical evaluations. Some of the results are part of the medical thesis of R. Engers. This work was supported by the Deutsche Forschungsgemeinschaft (Ga326/1-6) and by the Gesellschaff der Gonner und Forderer der Grundlagenforschung des Krebses.

**Figure 4** Growth curves of BA-HAN-1C in standard growth medium with 0.02% ethanol (control) and in medium supplemented with 1 μM retinoic acid. Each value represents the mean of three replicate samples ± standard deviation. The difference between the growth curves is statistically significant ($P<0.001$; analysis of variance with two independent factors). **a**: mean doubling time of 16 hours. **b**: mean doubling time of 17 hours.

### References

ALEXANDER, P. (1985). Do cancers arise from a single transformed cell or is monoclonality of tumors a late event in carcinogenesis? *Br. J. Cancer*, **51**, 453.

AMATRUDA, III, T.T., SIDELL, N., RANYARD, J. & KOEFFLER, H.P. (1985). Retinoic acid treatment of human neuroblastoma cells is associated with decreased n-myc expression. *Biochem. Biophys. Res. Comm.*, **126**, 1189.

ANNESLY, F., GIACHERIO, O. & WILKERSON, K. (1984). Analysis of retinoids by high performance liquid chromatography using programmed gradient separation. *J. Chromatogr.*, **305**, 199.

BENTLEY, D.L. & GROUDINE, M. (1986). A block to elongation is largely responsible for decreased transcription of c-myc in differentiated HL60 cells. *Nature*, **321**, 702.

BIESALKSI, H.K. & HAFNER, G. (1988). Trace analysis of retinyl esters by isocratic absorption HPLC. *J. Lipid Res.*, **55**, 255.

CHYTIL, F. (1986). Vitamin A: Its role in differentiation and development. In *Nutritional Diseases: Research Directions in Comparative Pathobiology*, p. 21. Alan R. Liss: New York.

CRAIG, R.W., MAUE, R.J., HROMCHAK, R.A. & BLOCH, A. (1984). Decline of c-myb expression in human myeloblastic leukaemia (ML-1) cells induced to differentiate with duanorubicin, conditioned medium, or retinoic acid. *Proc. Am. Assoc. Cancer Res.*, **25**, 64.

DELAPORTE, C., DAUTREAUX, B. & FARDEAU, M. (1986). Human myotube differentiation *in vitro* in different culture conditions. *Biol. Cell*, **57**, 17.

DE LARCO, I. & TODARO, G.J. (1978). Growth factors from murine sarcoma virus-transformed cells. *Proc. Natl Acad. Sci. USA.*, **75**, 4001.

DEXTER, D.L. (1977). *N,N*-dimethylformamide-induced morphological differentiation and reduction of tumorigenicity in cultured mouse rhabdomyosarcoma cells. *Cancer Res.*, **37**, 3136.

DOLLENMEIER, P. & EPPENBERGER, H.M. (1983). Differentiation of primary muscle cultured in a serum-free chemically defined medium. In *Hormonally Defined Media*, Fischer, G. & Wieser, R.J. (eds) p. 358. Springer: Berlin.

FLORINI, J.R., ROBERTS, A.B., EWTON, D.Z., FALEN, S.L., FLANDERS, K.C. & SPORN, M.B. (1986). Transforming growth factor-β. *J. Biol. Chem.*, **261**, 16509.

FRESHNEY, R.I. (1985). Induction of differentiation in neoplastic cells. *Anticancer Res.*, **5**, 111.

GABBERT, H.E., GERHARZ, C.D., ENGERS, R., MÜLLER-KLIESER, W. & MOLL, R. (1988). Terminally differentiated postmitotic tumor cells in a rat rhabdomyosarcoma cell line. *Virchows Arch. B*, **55**, 255.

GARVIN, A.J., STANLEY, W.S., BENNETT, D.D., SULLIVAN, J.J. & SENS, D.A. (1986). The *in vitro* growth, heterotransplantation, and differentiation of a human rhabdomyosarcoma cell line. *Am. J. Pathol.*, **125**, 208.

GERHARZ, C.D., GABBERT, H.E., MOLL, R., MELLIN, W., ENGERS, R. & GABBIANI, G. (1988). The intraclonal and interclonal phenotypic heterogeneity of a rhabdomyosarcoma cell line with abortive imitation of embryonic myogenesis. *Virchows Arch. B*, **55**, 193.

HARRIS, C.C., YOAKUM, G.H., LECHNER, J.F. & 5 others (1986). Growth, differentiation, and neoplastic transformation of human bronchial epithelial cells. In *Biochemical and Molecular Epidemiology of Cancer*, p. 213. Alan R. Liss: New York.

HELDIN, C.-H., WASTESON, A. & WESTERMARK, B. (1985). Platelet-derived growth factor. *Mol. Cell Endocrinol.*, **39**, 169.

HELDIN, C.-H. & WESTERMARK, B. (1986). Role of PDGF-like growth factors in autocrine stimulation of growth of normal and transformed cell. In *Oncogenes and Growth Control*, Kahn, P. & Graf, T. (eds) p. 43. Springer: Berlin.

JETTEN, A.M. (1980). Retinoids specifically enhance the number of epidermal growth factor receptors. *Nature*, **284**, 626.

KÖNIGSBERG, I.R. (1977). The role of the environment in the control of myogenesis *in vitro*. In *Pathogenesis of Human Muscular Dystrophies*, Rowland, L.P. (ed) p. 779. Excerpta Medica: Amsterdam.

LINDER, S., KROHNDAL, U., SENNERSTAM, R. & RINGERTZ, N.R. (1981). Retinoic acid-induced differentiation of F9 embryonal carcinoma cells. *Exp. Cell Res.*, **132**, 453.

LIPPMANN, S., KESSLER, J.F. & MEYSKENS JR., F.L. (1987*a*). Retinoids as preventive and therapeutic anticancer agents (part I). *Cancer Treat. Rep.*, **71**, 391.

LIPPMANN, S.M., KESSLER, J.F. & MEYSKENS JR., F.L. (1987*b*). Retinoids as preventive and therapeutic anticancer agents (part II). *Cancer Treat. Rep.*, **71**, 391.

LOTAN, R. (1980). Effects of vitamin A and its analogs (retinoids). On normal and neoplastic cells. *Biochim. Biophys. Acta*, **605**, 33.

MARQUARDT, H., HUNKAPILLER,. M.W., HOOD, L.E. & 4 others (1983). Transforming growth factors produced by retrovirus-transformed rodent fibroblasts and human melanoma cells: Amino acid sequence homology with epidermal growth factor. *Proc. Natl Acad. Sci. USA.*, **80**, 4684.

MOORE, E.E., MITRA, N.S. & MORITZ, E.A. (1986). Differentiation of F9 embryonal carcinoma cells. *Differentiation*, **31**, 183.

NADAL-GINARD, B. (1978). Commitment, fusion and biochemical differentiation of a myogenic cell line in the absence of DNA-synthesis. *Cell*, **15**, 855.

PAUKOVITS, J.B., PAUKOVITS, W.R. & LAERUM, O.D. (1986). Identification of a regulatory peptide distinct from normal granulocyte-derived hemoregulatory peptide produced by human promyelocytic HL-60 leukemia cells after differentiation induction with retinoic acid. *Cancer Res.*, **46**, 4444.

PIERCE, G.B. (1974*a*). The benign cells of malignant tumours. In *Developmental Aspects of Cancerogenesis and Immunity*, King, P.J. (ed) p. 3. Academic Press: New York.

PIERCE, G.B. (1974*b*). Neoplasms, differentiations and mutations. *Am. J. Pathol.*, **77**, 103.

PINSET, C. & WHALEN, R.G. (1983). Control of cell proliferation and differentiation in the myogenic cell line L6 by manipulation of culture conditions. In *Hormonally Defined Media*, Fischer, G. & Wieser, R.J. (eds) p. 396. Springer: Berlin.

SACHS, L. (1980). Constitutive uncoupling of pathways of gene expression that control growth and differentiation in myeloid leukemia: A model for the origin and progression of malignancy. *Proc. Natl Acad. Sci. USA.*, **77**, 6152.

SACHS, L. (1987). Cell differentiation and bypassing of genetic defects in the suppression of malignancy. *Cancer Res.*, **47**, 1981.

SCARPA, S., UHLENDORF, B.W. & CANTONI, G.L. (1975). The differentiation of L5/A10 myoblast cell line (a subclone of L5 line) is controlled by changes of culture conditions. *Cell Differentiation*, **17**, 105.

SHERMAN, M.I., GUBLER, M.L., BARKAI, U., HARPER, M.I., COPPOLA, G. & YUAN, J. (1985). Role of retinoids in differentiation and growth of embryonal carcinoma cells. In *Retinoids, Differentiation and Disease* (Ciba Foundation Symposium 113), Nugent, J. & Clark, S. (eds) p. 42. Pitman: London.

SHERMAN, M.I., EGLITIS, M.A. & THOMAS, R. (1986). Reversible and irreversible effects of retinol upon the phenotypic properties of embryonal carcinoma cells. *J. Embryol. Exp. Morphol.*, **93**, 179.

SIDELL, N., SARAFIN, T., KELLY, M., TSUCHIDA, T. & HAUSSLER, M. (1986). Retinoic acid-induced differentiation of human neuroblastoma: A cell variant system showing two distinct responses. *Exp. Cell Biol.*, **54**, 287.

SPORN, M.B. & ROBERTS, A.B. (1983). Role of retinoids in differentiation and carcinogenesis. *Cancer Res.*, **43**, 3034.

SPORN, M.B. & TODARO, G.J. (1980). Autocrine secretion and malignant transformation of cells. *N. Engl. J. Med.*, **303**, 878.

SPORN, M.B., ROBERTS, A.B., WAKEFIELD, L.M. & DE CROMBRUGGHE, B. (1987). Some recent advances in the chemistry and biology of transforming growth factor-beta. *J. Cell Biol.*, **105**, 1039.

STRICKLAND, S. & MAHDAVI, V. (1978). The induction of differentiation in teratocarcinoma stem cells by retinoic acid. *Cell*, **15**, 393.

THIELE, C.J., REYNOLDS, C.P. & ISRAEL, M.A. (1985). Decreased expression of n-myc precedes retinoic acid-induced morphological differentiation of human neuroblastoma. *Nature*, **313**, 404.

TODARO, G.J., FRYLING, C. & DE LARCO, J.E. (1980). Transforming growth factor produced by certain human tumors: Polypeptides that interact with epidermal growth factor receptors. *Proc. Natl Acad. Sci. USA.*, **77**, 5258.

YAFFE, D. & SAXEL, O. (1977). A myogenic cell line with altered serum requirements for differentiation. *Differentiation*, **7**, 159.

ZILE, M.H., CULLUM, M.E., SIMPSON, R.U., BARUA, A.B. & SWARTZ, D.A. (1987). Induction of differentiation of human promyelocytic leukemia cell line HL-60 by retinoyl glucuronide, a biologically active metabolite of vitamin A. *Proc. Natl Acad. Sci. USA.*, **84**, 2208.


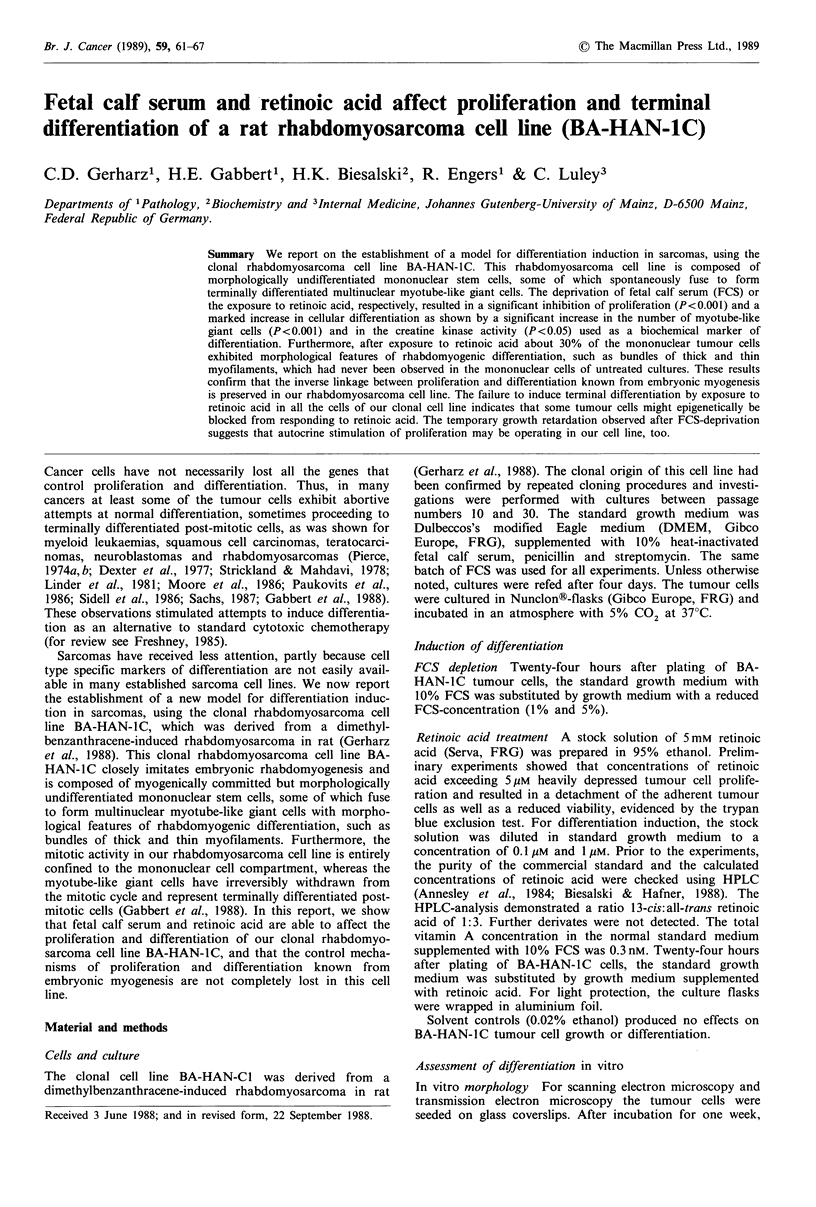

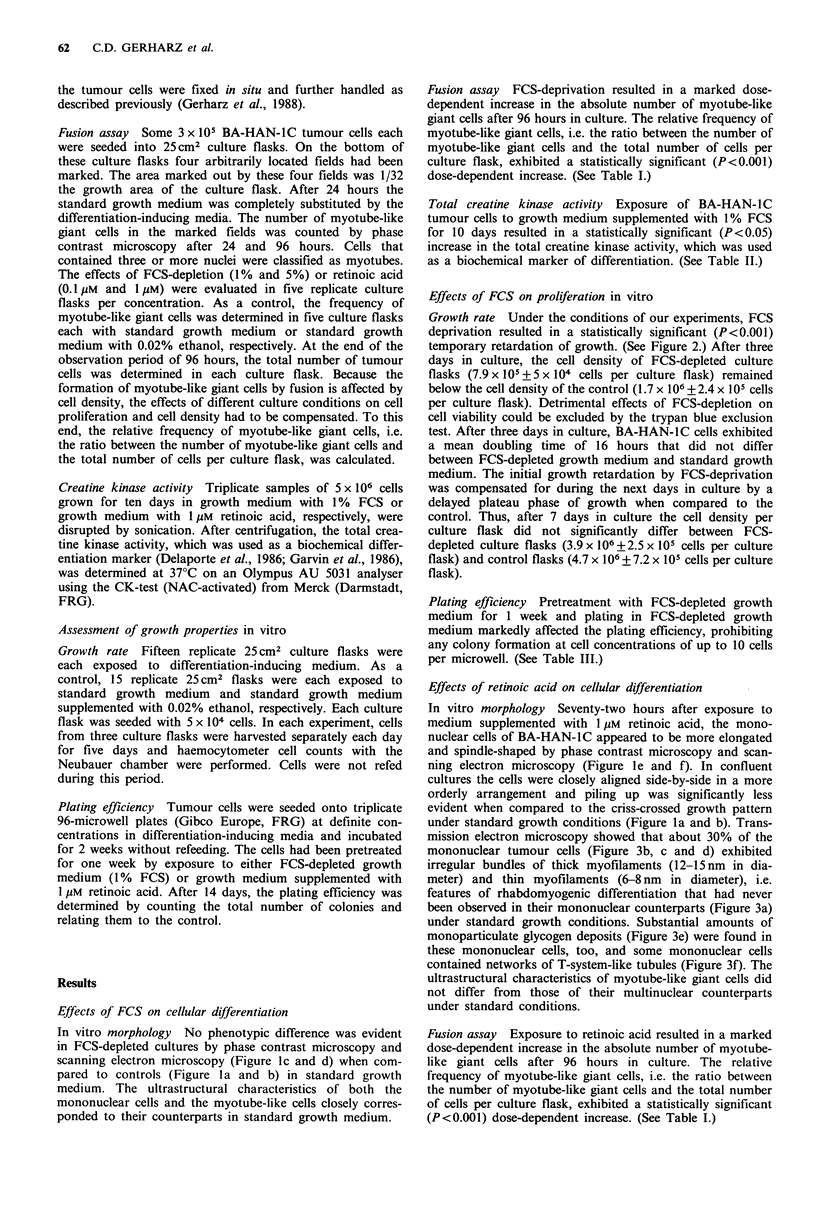

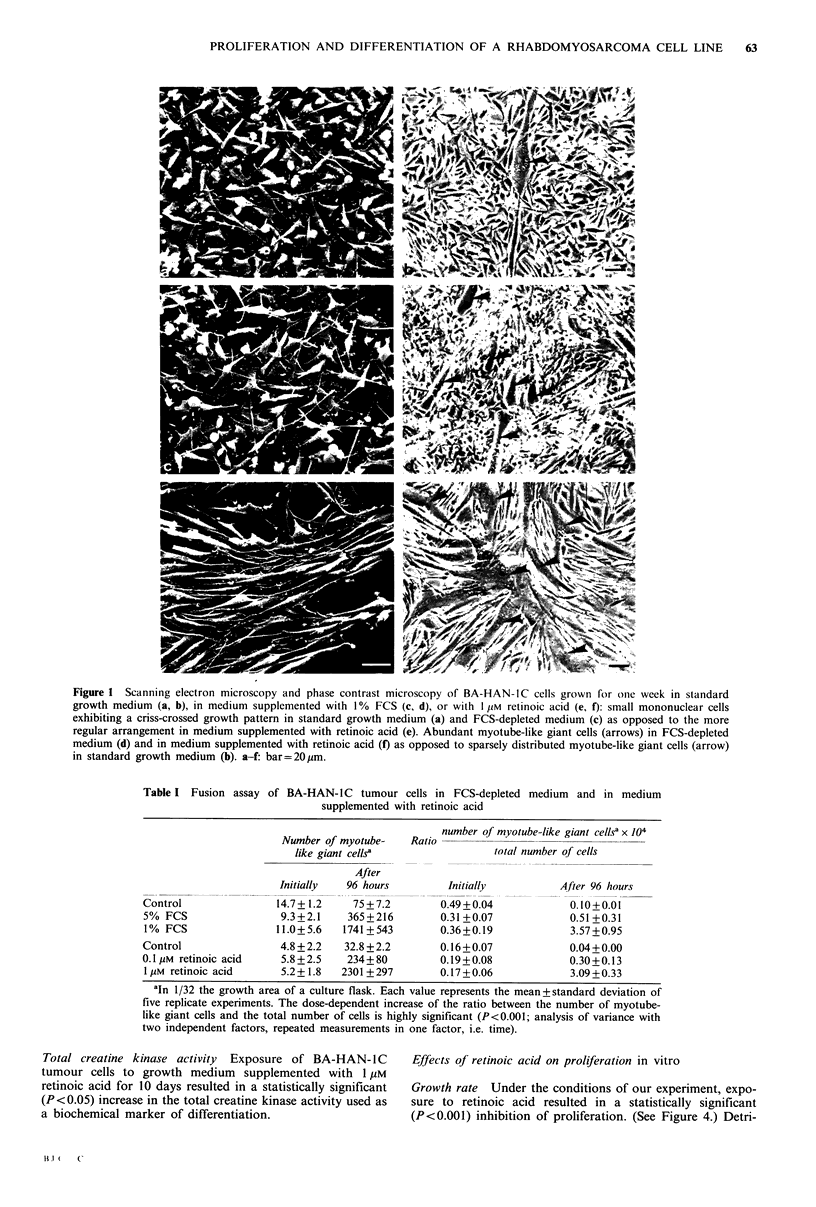

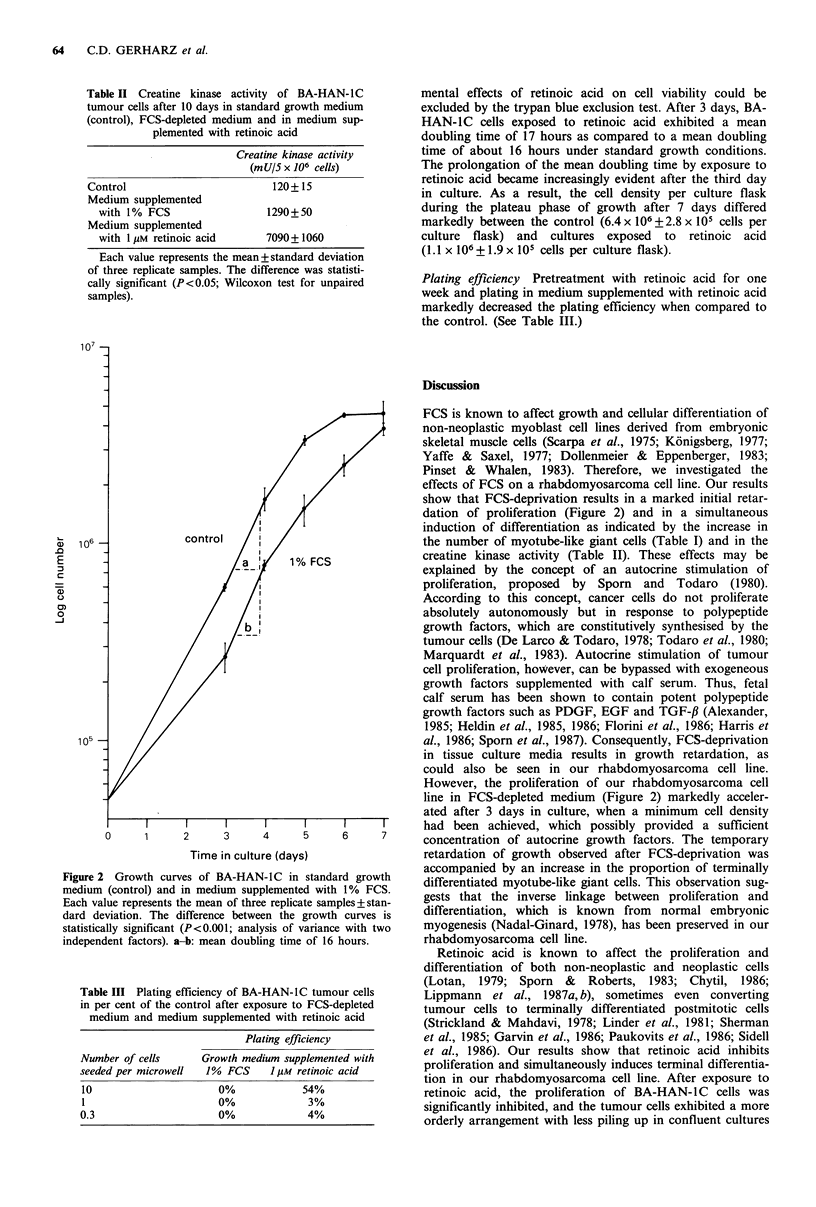

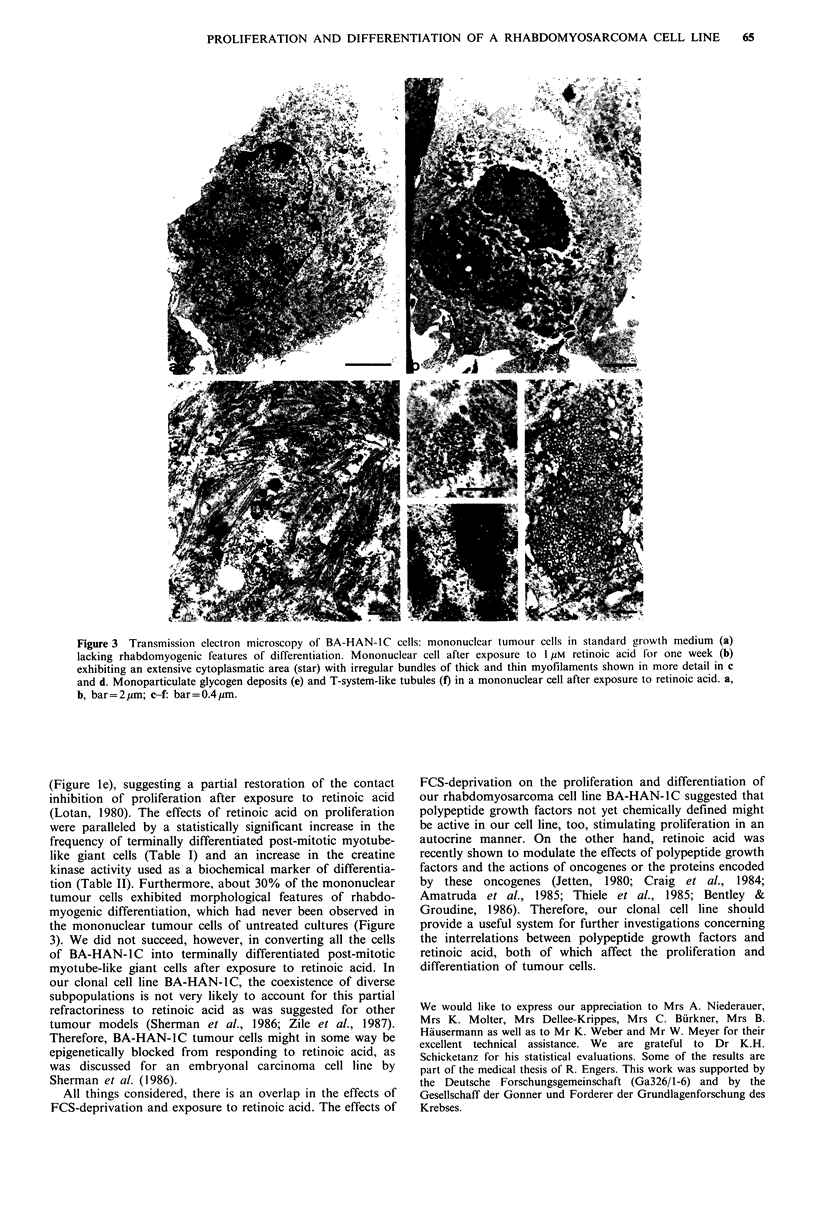

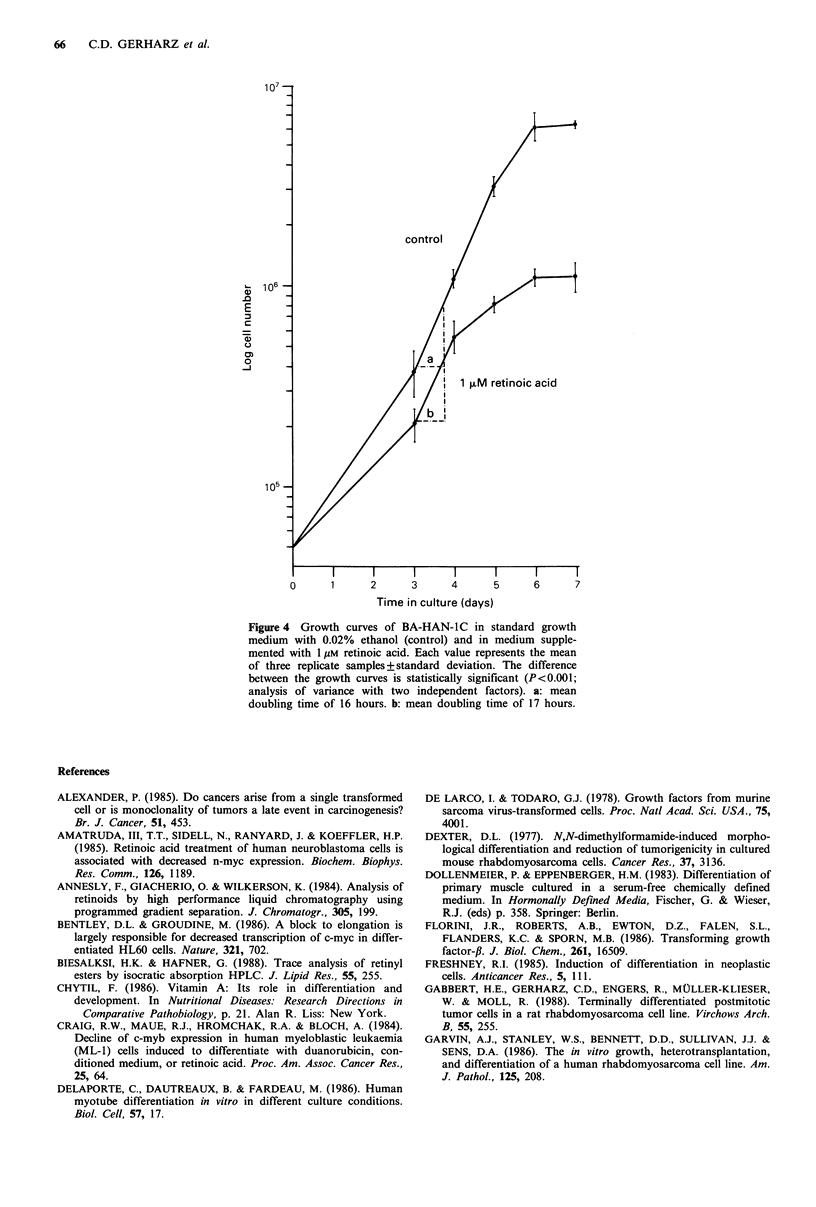

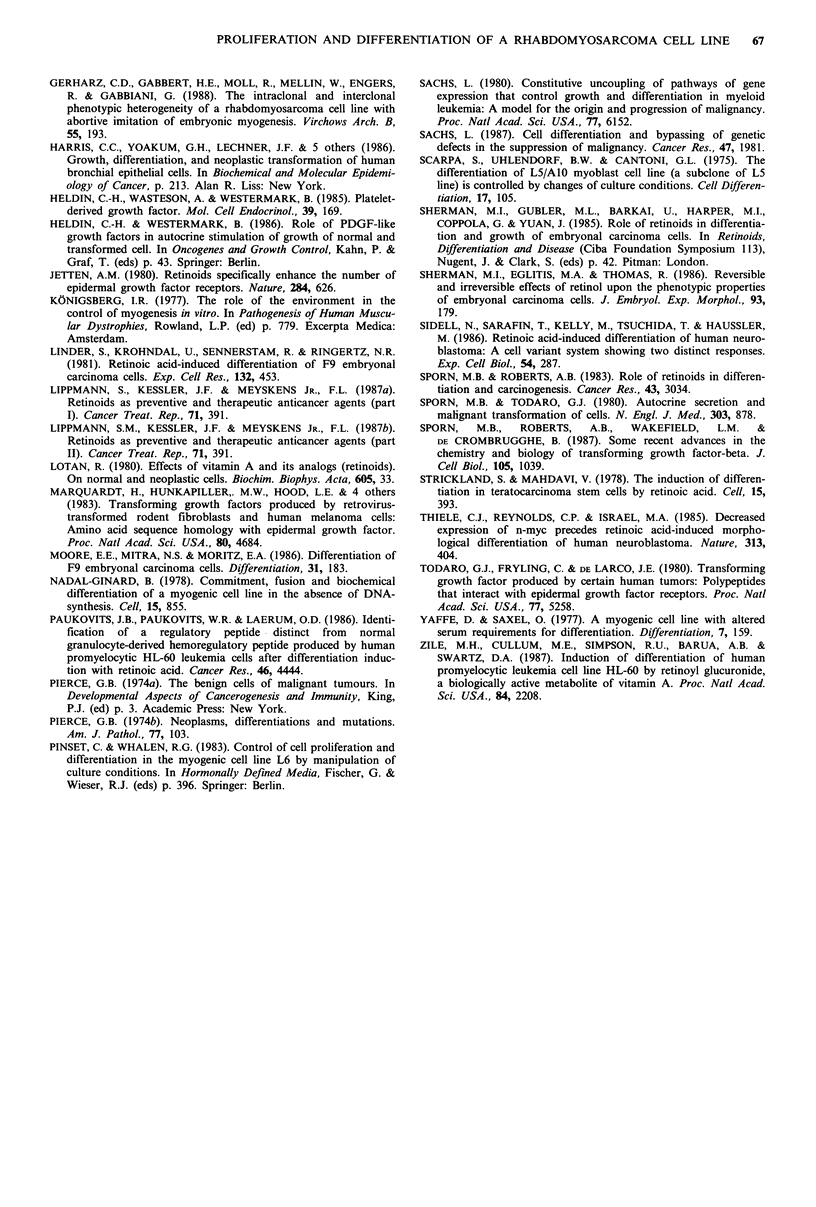


## References

[OCR_00553] Alexander P. (1985). Do cancers arise from a single transformed cell or is monoclonality of tumours a late event in carcinogenesis?. Br J Cancer.

[OCR_00558] Amatruda T. T., Sidell N., Ranyard J., Koeffler H. P. (1985). Retinoic acid treatment of human neuroblastoma cells is associated with decreased N-myc expression.. Biochem Biophys Res Commun.

[OCR_00564] Annesley T., Giacherio D., Wilkerson K., Grekin R., Ellis C. (1984). Analysis of retinoids by high-performance liquid chromatography using programmed gradient separation.. J Chromatogr.

[OCR_00569] Bentley D. L., Groudine M. (1986). A block to elongation is largely responsible for decreased transcription of c-myc in differentiated HL60 cells.. Nature.

[OCR_00591] Delaporte C., Dautreaux B., Fardeau M. (1986). Human myotube differentiation in vitro in different culture conditions.. Biol Cell.

[OCR_00601] Dexter D. L. (1977). N,N-Dimethylformamide-induced morphological differentiation and reduction of tumorigenicity in cultured mouse rhabdomyosarcoma cells.. Cancer Res.

[OCR_00612] Florini J. R., Roberts A. B., Ewton D. Z., Falen S. L., Flanders K. C., Sporn M. B. (1986). Transforming growth factor-beta. A very potent inhibitor of myoblast differentiation, identical to the differentiation inhibitor secreted by Buffalo rat liver cells.. J Biol Chem.

[OCR_00617] Freshney R. I. (1985). Induction of differentiation in neoplastic cells.. Anticancer Res.

[OCR_00621] Gabbert H. E., Gerharz C. D., Engers R., Müller-Klieser W., Moll R. (1988). Terminally differentiated postmitotic tumor cells in a rat rhabdomyosarcoma cell line.. Virchows Arch B Cell Pathol Incl Mol Pathol.

[OCR_00627] Garvin A. J., Stanley W. S., Bennett D. D., Sullivan J. L., Sens D. A. (1986). The in vitro growth, heterotransplantation, and differentiation of a human rhabdomyosarcoma cell line.. Am J Pathol.

[OCR_00635] Gerharz C. D., Gabbert H., Moll R., Mellin W., Engers R., Gabbiani G. (1988). The intraclonal and interclonal phenotypic heterogeneity in a rhabdomyosarcoma cell line with abortive imitation of embryonic myogenesis.. Virchows Arch B Cell Pathol Incl Mol Pathol.

[OCR_00648] Heldin C. H., Wasteson A., Westermark B. (1985). Platelet-derived growth factor.. Mol Cell Endocrinol.

[OCR_00658] Jetten A. M. (1980). Retinoids specifically enhance the number of epidermal growth factor receptors.. Nature.

[OCR_00668] Linder S., Krondahl U., Sennerstam R., Ringertz N. R. (1981). Retinoic acid-induced differentiation of F9 embryonal carcinoma cells.. Exp Cell Res.

[OCR_00673] Lippman S. M., Kessler J. F., Meyskens F. L. (1987). Retinoids as preventive and therapeutic anticancer agents (Part I).. Cancer Treat Rep.

[OCR_00683] Lotan R. (1980). Effects of vitamin A and its analogs (retinoids) on normal and neoplastic cells.. Biochim Biophys Acta.

[OCR_00686] Marquardt H., Hunkapiller M. W., Hood L. E., Twardzik D. R., De Larco J. E., Stephenson J. R., Todaro G. J. (1983). Transforming growth factors produced by retrovirus-transformed rodent fibroblasts and human melanoma cells: amino acid sequence homology with epidermal growth factor.. Proc Natl Acad Sci U S A.

[OCR_00693] Moore E. E., Mitra N. S., Moritz E. A. (1986). Differentiation of F9 embryonal carcinoma cells. Differences in the effects of retinoic acid, 5-bromodeoxyuridine, and N'-N'-dimethylacetamide.. Differentiation.

[OCR_00697] Nadal-Ginard B. (1978). Commitment, fusion and biochemical differentiation of a myogenic cell line in the absence of DNA synthesis.. Cell.

[OCR_00702] Paukovits J. B., Paukovits W. R., Laerum O. D. (1986). Identification of a regulatory peptide distinct from normal granulocyte-derived hemoregulatory peptide produced by human promyelocytic HL-60 leukemia cells after differentiation induction with retinoic acid.. Cancer Res.

[OCR_00714] Pierce G. B. (1974). Neoplasms, differentiations and mutations.. Am J Pathol.

[OCR_00730] Sachs L. (1987). Cell differentiation and bypassing of genetic defects in the suppression of malignancy.. Cancer Res.

[OCR_00724] Sachs L. (1980). Constitutive uncoupling of pathways of gene expression that control growth and differentiation in myeloid leukemia: a model for the origin and progression of malignancy.. Proc Natl Acad Sci U S A.

[OCR_00733] Scarpa S., Uhlendorf B. W., Cantoni G. L. (1985). The differentiation of L5/A10 myoblast cell line (a subclone of L5 line) is controlled by changes of culture conditions.. Cell Differ.

[OCR_00746] Sherman M. I., Eglitis M. A., Thomas R. (1986). Reversible and irreversible effects of retinol upon the phenotypic properties of embryonal carcinoma cells.. J Embryol Exp Morphol.

[OCR_00752] Sidell N., Sarafian T., Kelly M., Tsuchida T., Haussler M. (1986). Retinoic acid-induced differentiation of human neuroblastoma: a cell variant system showing two distinct responses.. Exp Cell Biol.

[OCR_00758] Sporn M. B., Roberts A. B. (1983). Role of retinoids in differentiation and carcinogenesis.. Cancer Res.

[OCR_00766] Sporn M. B., Roberts A. B., Wakefield L. M., de Crombrugghe B. (1987). Some recent advances in the chemistry and biology of transforming growth factor-beta.. J Cell Biol.

[OCR_00762] Sporn M. B., Todaro G. J. (1980). Autocrine secretion and malignant transformation of cells.. N Engl J Med.

[OCR_00772] Strickland S., Mahdavi V. (1978). The induction of differentiation in teratocarcinoma stem cells by retinoic acid.. Cell.

[OCR_00777] Thiele C. J., Reynolds C. P., Israel M. A. Decreased expression of N-myc precedes retinoic acid-induced morphological differentiation of human neuroblastoma.. Nature.

[OCR_00783] Todaro G. J., Fryling C., De Larco J. E. (1980). Transforming growth factors produced by certain human tumor cells: polypeptides that interact with epidermal growth factor receptors.. Proc Natl Acad Sci U S A.

[OCR_00789] Yaffe D., Saxel O. (1977). A myogenic cell line with altered serum requirements for differentiation.. Differentiation.

[OCR_00793] Zile M. H., Cullum M. E., Simpson R. U., Barua A. B., Swartz D. A. (1987). Induction of differentiation of human promyelocytic leukemia cell line HL-60 by retinoyl glucuronide, a biologically active metabolite of vitamin A.. Proc Natl Acad Sci U S A.

[OCR_00596] de Larco J. E., Todaro G. J. (1978). Growth factors from murine sarcoma virus-transformed cells.. Proc Natl Acad Sci U S A.

